# Case of Cholera—Unusually Protracted Suppression of Urine—Recovery

**Published:** 1866-12

**Authors:** N. S. Davis

**Affiliations:** Prof. Practical and Clinical Medicine, Chicago Medical College


### CASE OF CHOLERA—UNUSUALLY PROTRACTED SUPPRESSION OF URINE—RECOVERY.

By N. S. DAVIS, M.D., Prof. Practical and Clinical Medicine, Chicago Medical College.

Mr. P., a resident of Maine, but temporarily sojourning in this city, was attacked on the 28th of October, 1866, with diarrhœa, and had, during the day, three or four large serous discharges, but continued to attend to his business until evening. Between 6 and 7 o'clock in the evening, he had a very copious discharge accompanied by vomiting, and followed by partial syncope. Dr. Wilkins was called in at that time, and promptly applied sinapisms and external warmth freely, with several doses of medicine internally. The vomiting and purging, however, continued frequent and severe, and near 12 o'clock that night I was called to the patient, and requested by Dr. Wilkins to take charge of his further treatment. At that time, the patient presented every symptom of cholera in its active stage. The eyes were deeply sunken; the voice husky; skin cool and shrunken; pulse small and weak; the discharges frequent, copious in quantity, and having the appearance of slightly turbid water. When allowed to stand a little while, it deposited some mucus and epithelium at the bottom of the vessel. There were muscular cramps in the abdomen and lower extremities. Directions were given to have the sinapisms of mustard continued over the epigastrium and dorsal portion of the spine, with dry warmth to the feet and legs, a powder of calomel 1 gr., sulph. morph. $\frac{1}{4}$ gr., and acetate of lead $1\frac{1}{2}$ grs., to be given every hour; and a teaspoonful of an emulsion, consisting of chloroform ℨiij, sulph. morph. 3 grs., gum arabic and sugar each ℨiij, rubbed up with three ounces of mint-water, immediately after every turn of vomiting.

Oct. 29th, 8 o'clock A.M. Found the patient still vomiting, purging, and cramping, with no change since the night visit except increasing evidences of exhaustion. Learned, on inquiry, that the attendants had been so remiss as to have given less than half the amount of medicine directed at the previous visit; consequently, the same directions were repeated, with the addition of an enema after every discharge from the bowels, containing 10 grs. of acetate of lead and ½ gr. of acetate of morphia in half a teacupful of cold water.

12 o'clock M. Found the patient still vomiting violently, but no purging during the last two hours, and no cramps. The extremities are quite cold, corrugated, and blue; the pulse very small and weak; inclined to drowsiness between the paroxysms of vomiting, and has passed no urine since the evening of the 20th. As he seemed to reject immediately the chloroform emulsion, he was now directed to have a teaspoonful of the following solution immediately after every act of vomiting:—

| | | |
|---|---|---|
| ℞. | Acetis Plumbi, | 30 grs. |
| | Acetis Morph., | 3 grs. |
| | Acetic Acid, | 10 gtts. |
| | Aqua, | ℥iij |

Mix.

And to omit all other internal medicine. We also now began the work of trying to sustain the nervous sensibilities and replenish the exhausted elements of the blood, by giving tablespoonful doses of beef-tea, well salted, and strong coffee, alternately, every 15 minutes. 6 o'clock P.M. Vomiting ceased after the third dose of the solution of the acetate of lead and morphine, and no discharges of any kind since. No change in other symptoms since previous visit, except the patient is more drowsy. Continue the use of the beef-tea and coffee, without medicine. 10 o'clock P.M. No discharges of any kind, and no urine in the bladder. More warmth in the extremities; pulse slightly increased in volume, but still very weak; and more constant inclination to sleep, though easily aroused when spoken to. Directed the beef-tea to be given in doses of two tablespoonfuls at a time, and a solution of 3 drachms of acetas potass. in 3 ounces of water, of which a teaspoonful was to be given every hour.

Oct. 30th, 8 o'clock A.M. Patient much the same as at the preceding visit. There had been no evacuations of either fæces or urine; the mind was somnolent and a little incoherent; respiration slow; pulse soft and irregular; and skin nearly natural in temperature. Ordered a continuance of the beef-tea, and allowed tea to be drank in the place of coffee. Also directed the solution of acetate of potassa to be continued every two hours, and a teaspoonful of nitrous ether the intermediate hour. The patient was visited at 12 M., 6 P.M., and 10 P.M. The only change worthy of note was, one moderate evacuation from the bowels, of a semi-fluid consistence, dark green color, and offensive odor; but no secretion of urine. At the 10 o'clock P.M. visit, the hands were colder, and the pulse slower and weaker. The same treatment was continued, and 10 drops of tincture of cantharides added to each dose of the nitrous ether.

Oct. 31st, 8 o'clock A.M. Patient had remained through the night in a semi-comatose condition, with short periods of restlessness; breathing very slow and irregular; pulse of the same character; vessels of the conjunctiva injected; skin of the face dry and little above natural temperature, but extremities cool. Still no secretion of urine. As the symptoms of uremia were increasing, the use of acetas potassa, nitrous ether, and cantharides was abandoned, and 10 drops of oil of turpentine, with $\frac{1}{20}$ of a grain of strychnine, given every two hours, and as much beef-tea and sweet milk as the patient could be induced to take. In the afternoon, a dark, semi-fluid, and offensive evacuation from the bowels was procured by means of a large enema of warm salt water; and frictions with camphorated soap liniment and oil of turpentine were applied to the abdomen and spine. But no marked change took place in the symptoms of the patient.

Nov. 1st, 8 o'clock A.M. Condition of the patient still essentially the same. The respiration and circulation still slow, irregular, and intermitting; and the mind difficult to arouse from its lethargy. The attendants had not been able to induce him to swallow the emulsion of turpentine and strychnine, during the latter part of the night or this morning, and he had taken only a small quantity of milk and beef-tea. No evacuations had occurred, and the bladder was still empty. The attendants were directed to arouse his sensibilities by frequent application of cold water to his forehead and temples, and continue the effort to support him with milk, beef-tea, etc. I also directed a tablespoonful of the infusion of juniper berries to be given every hour, and cloths wet in warm water to be applied to the abdomen and lumbar region. At the visit at 9 o'clock P.M., finding it impossible to get enough of the infusion of juniper berries taken to produce any effect, and there appearing no material improvement in the condition of the patient, he was directed some gin diluted with sugar and water.

Nov. 2d, 8 o'clock A.M. Patient had some paroxysms of greater restlessness during the night. There is at present more heat in the forehead and face, but the extremities cooler; and no improvement either in the respiration or circulation. No evacuations, and the bladder still empty. Although the nurse had tried faithfully, he had not induced the patient to swallow more than four teaspoonfuls of the gin since it was ordered the previous evening. On account of the increased heat in the head, and the continued absence of all intestinal and urinary evacuations, I abandoned further efforts to give the gin, and directed a powder of calomel 2 grs. and nitrate of potassa 5 grs., to be given every two hours. 9 o'clock P.M. Has taken his powders regularly through the day; drank some milk and beef-tea; and had his head and face frequently bathed with cold water. Since 3 o'clock P.M., has become restless, with constant delirium, and a frequent desire to get out of bed. Between 7 and 8 o'clock, his efforts to get up were so persistent that the nurse assisted him up on his feet two or three times, and while thus on his feet, about 8 o'clock, he began to urinate directly on the floor, passing in all about a coffeecupful of urine, presenting a reddish brown color, and a strong urinous odor. During the last hour the patient has rested more quietly; there is less heat in his head; and the respiration and circulation are both perceptibly improved. We directed no change in his treatment, except that the powders should be continued at intervals of only once in four hours.

Nov. 3d, 8 o'clock A.M. Patient urinated twice during the night, passing nearly a half-pint of rather high-colored urine each time. He had also one rather large semi-fluid and dark-colored fæcal discharge. The mind is still bewildered, with frequent spells of restlessness and attempts to get out of bed; but in the intervals of quiet, his respiration is more uniform, though slow, and the pulse more steady. The free use of animal broth, milk, and tea was continued, and a teaspoonful of the following to be given every three hours:—

℞. Aromat. Sulph. Acid,------------------- ℥ij.

Syrup of Wild Cherry bark,------------- ℥ij.

Mix.

Nov. 4th, 8 o'clock A.M. He had urinated three times during the 24 hours, and had several dark-colored and semi-fluid evacuations from the bowels. His sleep had been interrupted by frequent periods of restlessness, and the mental faculties, instead of being dull, began to exhibit an undue degree of wakefulness and exhilaration. Both respiration and circulation have become more natural, but the forehead is still hot. On account of the increasing mental exhilaration, the prescription of the previous day was omitted, and, in its place, 10 grs. of bromide of potassa given every four hours; nourishment and drinks as before.

From this time, the patient continued to improve slowly, until his recovery was complete, although it was four or five days more before his mind became entirely free from hallucinations. It will be observed, that in this case the complete suppression of urine continued nearly five days, which is longer than I have ever seen before, followed by the recovery of the patient.

In one patient, attacked with cholera the present season, the urinary secretion was suppressed nearly three days, with all the symptoms of collapse, and yet recovery took place; but the convalescence was slow, and the lower half of the cornea in both eyes showed well-marked ulcerations.

